# Public and physicians’ support for euthanasia in people suffering from psychiatric disorders: a cross-sectional survey study

**DOI:** 10.1186/s12910-019-0404-8

**Published:** 2019-09-11

**Authors:** Kirsten Evenblij, H. Roeline W. Pasman, Agnes van der Heide, Johannes J. M. van Delden, Bregje D. Onwuteaka-Philipsen

**Affiliations:** 1 Department of Public and Occupational Health, Amsterdam Public Health research institute, Amsterdam UMC, Vrije Universiteit Amsterdam, P.O. Box 7057, 1007 MB Amsterdam, The Netherlands; 2 Department of Public Health, Erasmus MC, University Medical Center Rotterdam, Rotterdam, The Netherlands; 3 Department of Medical Humanities, Julius Center for Health Sciences and Primary Care, UMC Utrecht, Utrecht, The Netherlands

**Keywords:** Assisted suicide, Attitudes, Epidemiology, Euthanasia, Medical decision making, Psychiatric disorders, Public opinion

## Abstract

**Background:**

Although euthanasia and assisted suicide (EAS) in people with psychiatric disorders is relatively rare, the increasing incidence of EAS requests has given rise to public and political debate. This study aimed to explore support of the public and physicians for euthanasia and assisted suicide in people with psychiatric disorders and examine factors associated with acceptance and conceivability of performing EAS in these patients.

**Methods:**

A survey was distributed amongst a random sample of Dutch 2641 citizens (response 75%) and 3000 physicians (response 52%). Acceptance and conceivability of performing EAS, demographics, health status and professional characteristics were measured. Multivariable logistic regression analyses were performed.

**Results:**

Of the general public 53% were of the opinion that people with psychiatric disorders should be eligible for EAS, 15% was opposed to this, and 32% remained neutral. Higher educational level, Dutch ethnicity, and higher urbanization level were associated with higher acceptability of EAS whilst a religious life stance and good health were associated with lower acceptability. The percentage of physicians who considered performing EAS in people with psychiatric disorders conceivable ranged between 20% amongst medical specialists and 47% amongst general practitioners. Having received EAS requests from psychiatric patients before was associated with considering performing EAS conceivable. Being female, religious, medical specialist, or psychiatrist were associated with lower conceivability. The majority (> 65%) of the psychiatrists were of the opinion that it is possible to establish whether a psychiatric patient’s suffering is unbearable and without prospect and whether the request is well-considered.

**Conclusion:**

The general public shows more support than opposition as to whether patients suffering from a psychiatric disorder should be eligible for EAS, even though one third of the respondents remained neutral. Physicians’ support depends on their specialization; 39% of psychiatrists considered performing EAS in psychiatric patients conceivable. The relatively low conceivability is possibly explained by psychiatric patients often not meeting the eligibility criteria.

**Supplementary information:**

**Supplementary information** accompanies this paper at 10.1186/s12910-019-0404-8.

## Background

The Dutch euthanasia law (2002) is widely accepted by the general public and health care professionals in The Netherlands [1, 2]. In 2016, 88% of the Dutch general public supported the euthanasia law. In that same year, 57% of the Dutch physicians had at least once performed euthanasia or assisted suicide (EAS), and another 24% who had never performed EAS found it conceivable to do so in the future [3]. According to the euthanasia law, a person must be suffering unbearably from a medically classifiable disease to qualify for EAS. The law does not differentiate between somatic and psychiatric causes of suffering as long as the due care criteria are met [4, 5]. However, research has shown that physicians’ support for EAS *is* associated with cause of suffering [6, 7]. In 2011, 34% of the physicians could imagine performing EAS in patients with psychiatric disorders compared to over 80% in patients with cancer or another severe physical disease [7]. Of the general public, 28% found EAS acceptable in case of chronic depression [2]. In practice, 5% of EAS requests from people with psychiatric disorders were granted, corresponding to 1% of all 6585 reported EAS cases in the Netherlands [8, 9].

Recently, there has been much attention for EAS in people with psychiatric disorders. The increasing incidence of EAS requests by psychiatric patients and documentaries illustrating the lives and suffering of people with psychiatric disorders requesting EAS broadcasted on national television have given rise to public and political debate, both nationally and internationally [10–12]. This study aims to explore support of the public and physicians for EAS in people with psychiatric disorders and examine factors associated with acceptance and conceivability of performing EAS in these patients. The following research questions will be addressed: (i) does the general public consider EAS in patients with psychiatric disorders acceptable, (ii) do physicians consider performing EAS in these patients conceivable, and (iii) which demographic and health or professional characteristics are associated with acceptance and conceivability of performing EAS?

## Methods

### Design and participants

In the context of the third evaluation of the Dutch Euthanasia act, a cross-sectional study was conducted amongst the general public and physicians in the Netherlands. Data were collected between May and September 2016, during this time two reminders were sent. This study did not require review by an ethics committee under the Dutch Medical Research Involving Human Subjects Act, as it did not involve imposing any interventions or actions and no patients were involved [13].

#### General public

An online questionnaire was distributed amongst the members of the CentERpanel which comprises 2641 households that were randomly selected from the pool of national postal delivery addresses [14]. All members aged above 16 years were invited to complete our online questionnaire. Participants who did not fill out any question about EAS were excluded (*n* = 5). Demographic characteristics were provided by CentERpanel.

#### Physicians

A 12-page written questionnaire was sent to a random sample of 1100 general practitioners, 400 elderly care physicians, 1000 medical specialists (working in hospital) and 500 psychiatrists. Inclusion criteria were (i) having been working in adult patient care in the Netherlands for the last year and (ii) having a registered work or home address in the national databank of registered physicians (IMS Health). 343 physicians did not meet the criteria.

### Questionnaire

#### General public

Public acceptance of EAS in case of psychiatric suffering was operationalized as the level of agreement with following statement: “*I am of the opinion that patients with a psychiatric disorder should be eligible for EAS in case they ask for it”*, ranging from 1 (completely agree) to 5 (completely disagree). This statement was part of a longer list of statements assessing the opinions with regard to eligibility for EAS including other specific settings / medical conditions. We did not specify whether the psychiatric disorder was the main motivation for the EAS request. Although this is the most straightforward interpretation of the question in this context, there may have been some ambiguity as to whether the psychiatric suffering was secondary to a primary somatic condition leading to the EAS request.

Other measurements included demographics (gender, age, household composition, educational level, ethnicity, considering philosophy important, urbanization level living area), health status (perceived general health, presence of depression) and EAS-related characteristics (experience with a relative requesting for EAS, opinion about the Dutch euthanasia law, and knowing that people with psychiatric disorders are eligible for EAS). Using a vignette, respondents were also asked (i) whether they agreed with the performance of EAS by a physician in case of treatment-resistant depression in a middle-aged women, and (ii) whether they would ask for EAS themselves if they would be in the patient’s position (Additional file 1: Figure S1).

#### Physicians

All physicians were asked whether they found it conceivable (yes/no) that they would ever perform EAS in patients with psychiatric disorders. This specification, “in patients with psychiatric disorders”, was omitted for psychiatrists as they presumably do not receive primary EAS requests from patients without psychiatric disorders. In the Netherlands, patients requesting EAS without a psychiatric disorder will usually not discuss their primary request with a psychiatrist, but rather with their general practitioner, although a psychiatrist might be consulted as a second opinion. Other measurements included demographics (gender, age, religious beliefs) and professional characteristics (specialty, years of experience, having completed palliative care training, being a palliative care consultant, being trained as independent consultant for the EAS procedure (SCEN-physician), ever having received/granted an EAS request either or not from patients with psychiatric disorders). Eight additional questions concerning opinions regarding EAS in people with psychiatric disorders were added to the questionnaire for psychiatrists.

### Statistical analyses

#### General public

First, univariable logistic regression analyses were performed to analyze which factors (demographics and health status) were associated with public acceptance of EAS. The five-point Likert Scale was dichotomized into EAS acceptable (agree or completely agree) and EAS not acceptable or neutral (disagree, completely disagree, and neutral). Next, all demographic and health factors were included in multivariable analysis. Manual stepwise backward selection (removal at *p* > 0.10) was performed to identify the variables strongest associated with public acceptance of EAS. Odds ratios (ORs) with 95% confidence intervals (CIs) were calculated.

#### Physicians

To analyse which demographics and professional factors were associated with conceivability of performing EAS an identical approach was applied starting with univariable logistic regression analyses followed by multivariable analyses using a manual stepwise backward selection. The variables ‘SCEN-physician’ and ‘ever having received/granted a request’ were not entered into the multivariable model due to collinearity with other variables in the model (age and specialty).

## Results

A total of 1965 eligible CentERpanel members (74%) responded. Table 1 provides an overview of their background characteristics. Of the respondents, 50.5% were male and 20.7% were 70 years or older. The majority (73.6%) were living with a partner and were Dutch (97.7%). Most (82.7%) respondents perceived their health to be (very) good and 4 % had a depression (self-reported). Of the people who supported the euthanasia law, 76.4% could imagine to request EAS themselves. A substantial group (38.1%) did not know whether or not psychiatric patients are eligible for EAS and 27.3% incorrectly assumed that they are not.

**Table 1:** Background characteristics of the respondents to the online survey (*n* = 1965)^a^

	No.	%
Demographics		
Gender		
Male	992	50.5
Female	973	49.5
Age (years)		
16–39	414	21.1
40–69	1144	58.2
≥ 70	407	20.7
Composition household		
Living with partner	1446	73.6
Living without partner	519	26.4
Education		
Low	552	28.1
Middle	636	32.4
High	777	39.5
Ethnicity		
Dutch	1897	97.7
Non-Dutch	45	2.3
Belongs to a philosophic sector		
Yes	954	49.2
No	984	50.8
Considers philosophy important		
Yes	378	19.2
No philosophy / philosophy not important	1587	80.8
Urbanization living area		
Low urban	759	39.0
Middle urban	402	20.7
High urban	783	40.3
Health status		
General health		
(very) good	1626	82.7
Moderate – (very) bad	339	17.3
Presence depression		
Yes	79	4.0
No	1886	96.0
Characteristics related to EAS		
Experience: someone close has requested a physician for EAS		
Yes	657	33.5
No	1305	66.5
Opinion: Do you think it is a good thing that there is an euthanasia law		
Yes, I reckon I would request EAS	1498	76.4
Yes, but I would never request EAS myself	241	12.3
No, I do not think it is good to have this law	14	0.7
No, I am against EAS	99	5.0
Do not know	110	5.6
Knowledge: Psychiatric patients are not eligible for EAS (incorrect).		
Agree (incorrectly answered)	536	27.3
Disagree (correctly answered)	681	34.7
Do not know	748	38.1

^a^ Number of missing varied between 0 and 27 (1.4%)

Of the 2657 eligible physicians, 1374 responded (52%). 20 were excluded because their specialty was unknown. The respondents’ background characteristics are shown in Table 2. 78% of general practitioners, 47.8% of elderly care physicians, 22.7% of medical specialists and 3.7% of psychiatrists had ever performed EAS in general, i.e. not specifically in people with psychiatric disorders. 16.3% of the psychiatrists had received EAS requests from one or more patients with psychiatric disorders in the last year. General practitioners, elderly care physicians, and medical specialists were less likely (0.6–4.6%) to have received such a request.

**Table 2:** Background characteristics of physicians (*n* = 1354)^a^

	General practitioners	Elderly care physicians	Medical specialists	Psychiatrists
	*N* = 607	*N* = 209	*N* = 331	*N* = 207
	No. (%)	No. (%)	No. (%)	No. (%)
Demographics				
Gender				
Male	260 (43.3)	80 (38.5)	198 (60.0)	122 (59.5)
Female	341 (56.7)	128 (61.5)	132 (40.0)	83 (40.5)
Age in years				
< 40	167 (27.5)	28 (13.4)	88 (26.6)	20 (9.6)
40–54	280 (46.1)	105 (50.2)	176 (53.2)	103 (49.8)
≥ 55	160 (26.4)	76 (36.4)	67 (20.2)	84 (40.6)
Religious belief				
No	398 (66.6)	130 (62.5)	241 (73.7)	117 (57.9)
Yes	200 (33.4)	78 (37.5)	86 (26.3)	85 (42.1)
Professional characteristics				
Years of experience				
< 10	142 (23.4)	22 (10.5)	65 (19.6)	46 (22.2)
≥ 10	465 (76.6)	187 (89.5)	266 (80.4)	161 (77.8)
Palliative care education				
No	261 (43.6)	76 (36.9)	257 (77.9)	195 (96.1)
Yes	338 (56.4)	130 (63.1)	73 (22.1)	8 (3.9)
Consultant palliative care/member palliative care team				
No	597 (98.5)	181 (87.9)	309 (93.9)	202 (99.5)
Yes	9 (1.5)	25 (12.1)	20 (6.1)	1 (0.5)
SCEN physician				
No	580 (95.7)	194 (94.2)	325 (99.1)	199 (98.0)
Yes	26 (4.3)	12 (5.8)	3 (0.9)	4 (2.0)
Ever received an explicit EAS request				
No	42 (6.9)	49 (23.4)	182 (55.2)	111 (58.1)
Yes, but never performed EAS	92 (15.2)	60 (28.7)	73 (22.1)	73 (38.2)
Yes, and ever performed EAS	472 (77.9)	100 (47.8)	75 (22.7)	7 (3.7)
Received an EAS request from a psychiatric patient in the past year				
No	564 (95.4)	196 (95.6)	325 (99.4)	164 (83.7)
Yes	27 (4.6)	9 (4.4)	2 (0.6)	32 (16.3)
Ever performed EAS on request from a psychiatric patient^b^				
Nee	572 (95.2)	199 (95.2)	327 (99.1)	184 (96.3)
Ja	29 (4.8)	10 (4.8)	3 (0.9)	7 (3.7)

^a^ Number of missing varied between 0 (0%) and 35 (2.6%)

^b^ General practitioners, medical specialists and elderly care physicians were asked whether they found it conceivable that they would perform EAS in patients with psychiatric disorders. This specification, ‘in patients with psychiatric disorders’, was omitted for psychiatrists, as they presumably do not receive EAS requests from patients without psychiatric disorders

### The acceptability and conceivability of EAS in people with psychiatric disorders

**Fig. 1:**
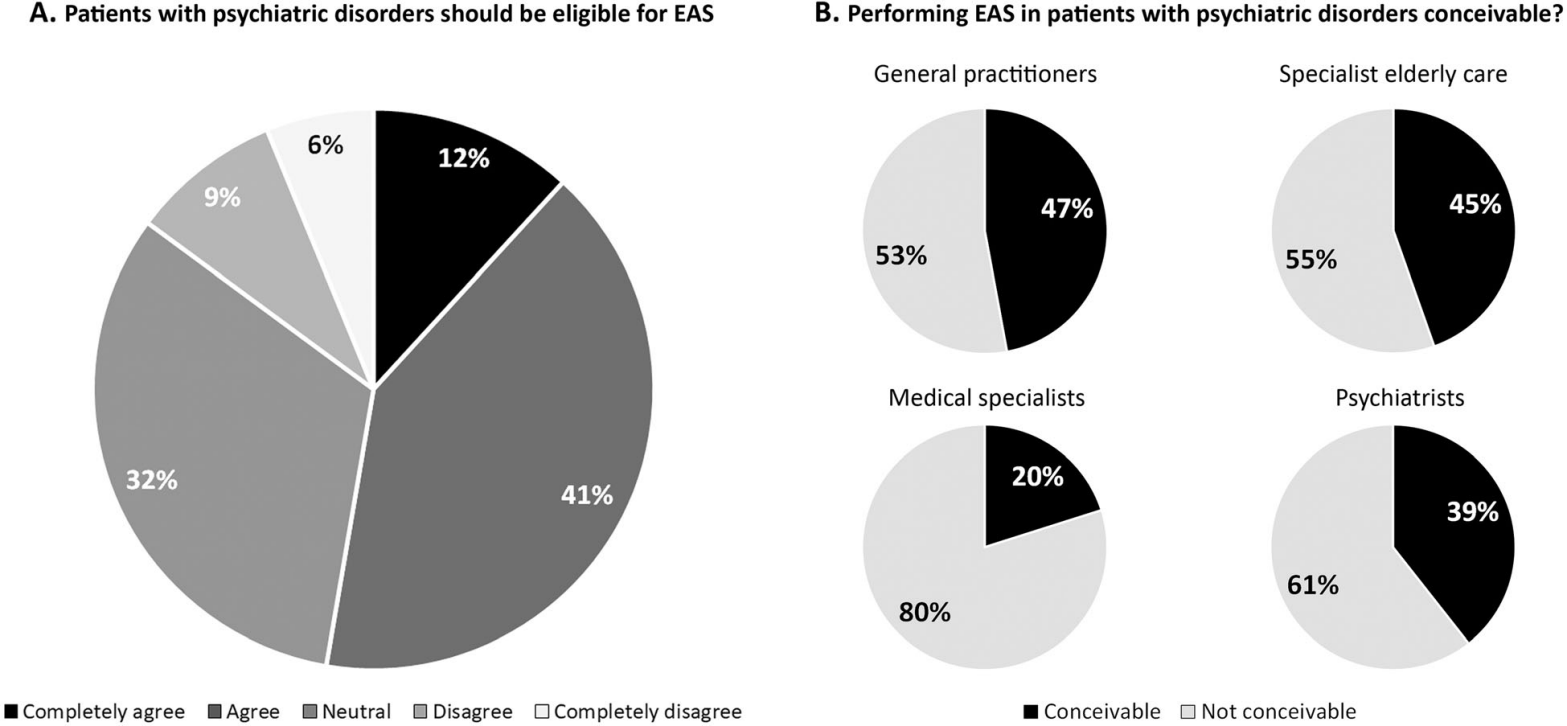
Public acceptance of EAS in people with psychiatric disorders and physician’s conceivability of performing EAS in these patients.* *General public: 19 missing (1.0%), physicians: 109 missing (8.1%). General practitioners, medical specialists and elderly care physicians were asked whether they found it conceivable that they would perform EAS in patients with psychiatric disorders. This specification, ‘in patients with psychiatric disorders’, was omitted for psychiatrists, as they presumably do not receive EAS requests from patients without psychiatric disorders

Just over half of the general public (53%) were of the opinion that people with psychiatric disorders should be eligible for EAS in case they ask for it (Figure 1) and 15% strongly opposed this. Reviewing the hypothetical case of a middle-aged patient with treatment-resistant depression who asks her psychiatrist for physician-assisted suicide, 39% of the general public agreed with providing EAS in this case, 31% did not know whether or not to agree and 30% disagreed. 29% reported that they would ask a physician to end their own life if they would be in the patient’s position, 20% would not and 51% did not know (Additional file 1: Figure S1). Of the physicians, general practitioners were most likely to consider performing EAS in people with psychiatric disorders conceivable (47%), followed by elderly care physicians (45%) and psychiatrists (39%) (Fig. 1). Medical specialists were least likely to find it conceivable (20%).

### Determinants of public acceptance of EAS in case of psychiatric suffering

In univariable analyses age, educational level, ethnicity, importance of religious life-stance, and urbanization level showed significant associations with acceptance of EAS in case of psychiatric suffering (Table 3). The multivariable model showed that respondents who had a middle (OR 1.292 [1.017–1.641]) or higher educational level (OR 1.914 [1.517–2.416]), a Dutch ethnicity (OR 1.958 [1.026–3.736]), or who were living in a highly urbanized area (OR 1.267 [1.024–1.567]) were more likely to accept EAS. Those who deemed their religious life-stance important (OR 0.276 [0.215–0.354]) or who had a (very) good health (OR 0.754 [0.586–0.969]) were less likely to accept it.

**Table 3:** Characteristics associated with the general public acceptability of EAS in case of a psychiatric condition (*n* = 1946)*

	Absolute	EAS acceptable	Univariable	Multivariable
	numbers	Row %	OR (95% CI)	OR (95% CI)
Gender				
Male	983	50.8	Reference	
Female	963	54.6	1.167 (0.977–1.395)	–
Age				
16–39	408	54.2	**1.400 (1.062–1.846)**	
40–69	1136	54.6	**1.424 (1.133–1.789)**	
≥ 70	402	45.8	Reference	–
Living with partner				
No	510	54.7	Reference	
Yes	1436	51.9	0.895 (0.731–1.096)	–
Education level				
Low	551	44.3	Reference	Reference
Middle	624	50.5	**1.283 (1.019–1.614)**	**1.292 (1.017–1.641)**
High	771	60.4	**1.922 (1.540–2.399)**	**1.914 (1.517–2.416)**
Ethnicity				
Non-Dutch	45	37.8	Reference	Reference
Dutch	1897	53.0	**1.856 (1.009–3.413)**	**1.958 (1.026–3.736)**
Religious life-stance important				
No	1568	58.7	Reference	Reference
Yes	378	27.5	**0.267 (0.208–0.341)**	**0.276 (0.215–0.354)**
Urbanization level				
Low	751	49.1	Reference	Reference
Middle	399	50.6	1.062 (0.833–1.353)	1.075 (0.833–1.386)
High	775	57.3	**1.389 (1.135–1.699)**	**1.267 (1.024–1.567)**
General health				
Less than good	334	57.2	Reference	Reference
(very) Good	1612	51.7	0.803 (0.633–1.018)	**0.754 (0.586–0.969)**
Presence of depression				
No	1868	52.4	Reference	
Yes	78	59.0	1.305 (0.824–2.068)	–

* Number of missing varied between 0 and 21 (1.1%). Bold indicates statistical significance (*p* < 0.05)

– indicates the item was entered in the regression but was eliminated in the stepwise procedure because *p* > 0.10

### Determinants of physicians’ conceivability of performing EAS

In univariable analyses age, religion, specialty, palliative care training, SCEN-physician, having performed EAS, and having received an EAS request from a psychiatric patient in the last year showed significant associations with conceivability of performing EAS in case of psychiatric suffering (Table 4). The multivariable model showed that physicians who had received EAS requests from psychiatric patients were more likely to find performing EAS conceivable (OR 1.828 [1.074–3.113]). Physicians who were female (OR 0.769 [0.599–0.988]), religious (OR 0.419 [0.320–0.549]), a medical specialist (OR 0.242 [0.171–0.340]), or a psychiatrist (OR 0.651 [0.455–0.932]) were less likely to find it conceivable.

**Table 4:** Characteristics associated with the physician’s conceivability of performing EAS in case of psychiatric suffering (*n* = 1245)*

	Absolute	EAS conceivable	Univariable	Multivariable
	numbers	Row %	OR (95% CI)	OR (95% CI)
Gender				
Male	607	39.4	Reference	Reference
Female	629	37.7	0.931 (0.740–1.171)	**0.769 (0.599–0.988)**
Age				
< 40	288	40.3	0.863 (0.628–1.186)	–
40–54	615	35.0	**0.688 (0.525–0.902)**	–
≥ 55	342	43.9	Reference	
Religious beliefs				
No	802	44.3	Reference	Reference
Yes	425	27.5	**0.478 (0.371–0.617)**	**0.419 (0.320–0.549)**
Specialty				
General practitioner	535	47.1	Reference	Reference
Elderly care physician	193	44.6	0.903 (0.648–1.256)	0.954 (0.675–1.346)
Medical specialist	314	20.1	**0.282 (0.204–0.390)**	**0.242 (0.171–0.340)**
Psychiatrist	203	39.4	0.730 (0.526–1.015)	**0.651 (0.455–0.932)**
Years of experience				
< 10	265	35.8	Reference	
≥ 10	980	39.4	1.163 (0.877–1.541)	–
Completed palliative care training				
No	742	36.1	Reference	
Yes	489	41.9	**1.277 (1.010–1.613)**	–
SCEN physician				
No	1197	36.8	Reference	
Yes	39	89.7	**15.000 (5.296–42.485)**	NE
Consultant palliative care/member palliative care team				
No	1186	38.3	Reference	
Yes	50	44.0	1.267 (0.716–2.241)	–
Ever received an EAS request				
No	378	23.0	Reference	
Yes, but never performed EAS	292	27.7	1.284 (0.904–1.823)	NE
Yes, and performed EAS	561	54.4	**3.985 (2.977–5.334)**	NE
Received an EAS request from a psychiatric patient in the past year				
No	1148	37.3	Reference	Reference
Yes	67	55.2	2.075 (1.263–3.408)	**1.828 (1.074–3.113)**

* Number of missing varied between 0 and 30 (2.4%). The variables ‘SCEN-physician’ and ‘ever having received/granted a request’ were not entered into the multivariable model due to collinearity with other variables in the model. Bold indicates statistical significance (*p* < 0.05)

– indicates the item was entered in the regression but was eliminated in the stepwise procedure because *p* > 0.10, NE indicates the item was not entered in the regression

### The opinion of psychiatrists with regard to EAS in people with psychiatric disorders

The majority of the psychiatrists were of the opinion that it is possible to assess whether a psychiatric patient’s suffering is unbearable and without prospect of improvement (69.8%), and to establish whether a wish to die of a psychiatric patient is well-considered or the consequence of an underlying pathology (65.4%) (Additional file 2: Table S1). These two assessments are part of the due care criteria, determining a patient’s eligibility for EAS. Also, 64.2% stated that providing assistance with suicide is compatible with a care provider relationship. Twenty-two percent of the psychiatrists were of the opinion that when deciding whether or not to grant a request, psychiatrists need to take account of the possibility that an effective therapeutic treatment might become available in future, and one fourth were of the opinion that physician assisted-suicide should not be used to prevent suicide.

## Discussion

Our results reveal that 53% of the general public were of the opinion that people suffering from psychiatric disorders should be eligible for EAS. Higher educational level, Dutch ethnicity, and higher urbanization level were associated with higher acceptance of EAS whilst a religious life stance and good health were associated with lower acceptance. Less than half of the physicians considered it conceivable to perform EAS in people with psychiatric disorders, especially psychiatrists and medical specialists showed restraint. Having received EAS requests from psychiatric patients before was associated with higher conceivability of performing EAS in case of psychiatric suffering. Being female, religious, medical specialist, or psychiatrist were associated with lower conceivability.

Comparing our results with results of a previous study (2010) using the same vignette demonstrates that the percentage of the general public supporting EAS in case of treatment-resistant depression has increased from 28% in 2010 [2] to 40% in 2016. Compared with 2010, general practitioners’ conceivability of performing EAS in case of psychiatric suffering increased as well, though the conceivability of medical specialists and elderly care physicians remained the same [7]. Our findings are in line with an (inter) national trend towards acceptance of EAS in general [2, 15–18]. Surprisingly, psychiatrists’ conceivability of performing EAS decreased, from 47% in 1995 [19] to 39% in 2016. The establishment of the End-of-life clinic in 2012 may have contributed to this [11]. The clinic, which works with mobile teams of qualified physicians and nurses, was founded to provide EAS to patients who meet the statutory due care criteria but whose own physician does not feel competent or feels reluctant to provide EAS. Psychiatrists might be more inclined to give in to their reluctance to perform EAS now they can refer their patients to the End-of-life clinic. It is also possible that the increasing number of EAS requests to psychiatrists caused them to ponder their own position and made them more aware of the medical and ethical difficulties in this delicate matter resulting in more reluctance.

Corroborating the results of a previous study, this study showed that physicians’ specialty was associated with the conceivability of performing EAS in case of psychiatric suffering [7]. Medical specialists and psychiatrists were significantly less likely to consider performing EAS, possibly because they have less experience with EAS in general; i.e. they receive fewer requests and less frequently perform EAS [3]. The low conceivability among psychiatrists to perform EAS may also be related to their opinions regarding EAS in psychiatric practice. Based on our results, psychiatrists’ reticence to perform EAS seems to be caused neither by the conviction that assessing the capacity and suffering of patients is impossible, nor by the conviction that providing EAS is incompatible with a psychiatric care relationship. Their reticence rather may be explained by doubts about whether or not there still is prospect of improvement [9]. The unpredictability of the course and prognosis of psychiatric disorders and the large variety of treatment options for psychiatric disorders make it difficult to establish that there are no other reasonable treatment alternatives and that euthanasia is indeed the only option [20–23]. In addition, our results showed that one fifth of psychiatrists was of the opinion that psychiatrists should consider the possibility that effective treatment might become available in the future when deciding whether or not to grant a request. The Dutch Euthanasia Code and the guideline of the Dutch Association, however, do not require physicians to consider this solely theoretical possibility, but state that treatment should be effective ‘within the foreseeable future’. [23, 24]

### Strengths and limitations

The most important strengths of this study are the nationwide sample of the general public and physicians, representing different specialties, and the substantial response, especially of the general public. A possible limitation is selection bias. Although the CentERpanel aims to be representative of the Dutch general public, a comparison with the Dutch population register data of Statistics Netherlands [25] showed that the study participants were slightly older and higher educated, and that migrants were underrepresented. Furthermore, it is possible that the interpretation of the concept of ‘conceivability’ caused bias. Medical specialists may simply not consider it conceivable to perform EAS in patients whose suffering is solely of psychiatric nature because they are not responsible for the care of these patients. However, this does not hold for psychiatrists. Their reticence is most likely based on substantive reasons.

## Conclusion

The general public shows more support (53%) than opposition (15%) as to whether patients suffering from a psychiatric disorder should be eligible for EAS, even though one third of the respondents remained neutral. Physicians’ support depends on their specialization. General practitioners and elderly care specialists are most positive; about half considers performing EAS conceivable. Fewer medical specialists (20%) and psychiatrist (39%) consider performing EAS conceivable. Although, over the years, conceivability increased for general practitioners and remained stable for medical specialists and elderly care specialists, it decreased amongst psychiatrists. As the majority of the psychiatrists were of the opinion that it is possible to establish whether a psychiatric patient’s suffering is unbearable and without prospect and whether the request is well-considered, the relatively low conceivability of performing EAS is possibly explained by psychiatric patients often not meeting the eligibility criteria as has been shown previously [9].

## Supplementary information

**Additional file 1: Figure S1.** Vignette: Mrs Langezaal is middle-aged. She is physically well, but mentally ill. She has been suffering from severe depression for years and her psychiatrist’s treatment has not worked. She regularly tells her physicians that she wants to die. She already has had one unsuccessful suicide attempt. Mrs Langezaal visits her psychiatrist and asks for physician-assisted suicide. The psychiatrist decides to honour her request and performs physician-assisted suicide.* * The general public was asked to reflect on this vignette. Number of missings: 22 (1.1%). (JPG 295 kb)

**Additional file 2: Table S1.** Opinions of psychiatrists with regard to EAS in people with psychiatric disorders. (DOCX 18 kb)

## Data Availability

The datasets used and/or analyzed during the current study are available from the corresponding author on reasonable request.

## Abbreviations

EAS: Euthanasia and assisted suicide
SCEN-physician: A physician who is trained as independent consultant for the EAS procedure (SCEN: Support and Consultation on Euthanasia in the Netherlands)
