# Supplementary figures and images for: Public and physicians’ support for euthanasia in people suffering from psychiatric disorders: a cross-sectional survey study
Source: BMC Med Ethics. 2019 Sep 11;20:62. doi: 10.1186/s12910-019-0404-8 (PMC6737595; doi:10.1186/s12910-019-0404-8)

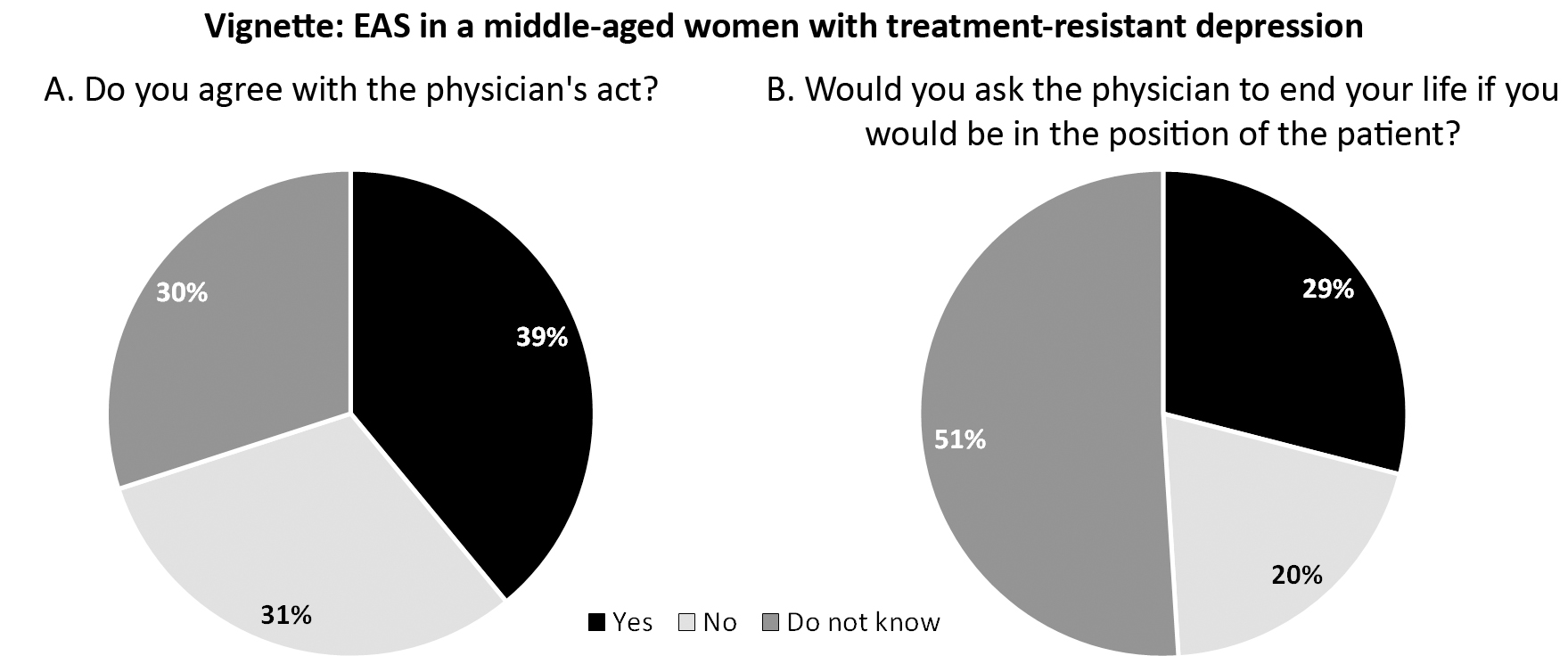

Supplement: Supplementary file 1 — Additional file 1: Figure S1. Vignette: Mrs Langezaal is middle-aged. She is physically well, but mentally ill. She has been suffering from severe depression for years and her psychiatrist’s treatment has not worked. She regularly tells her physicians that she wants to die. She already has had one unsuccessful suicide attempt. Mrs Langezaal visits her psychiatrist and asks for physician-assisted suicide. The psychiatrist decides to honour her request and performs physician-assisted suicide.* * The general public was asked to reflect on this vignette. Number of missings: 22 (1.1%). (JPG 295 kb) [file 12910_2019_404_MOESM1_ESM.jpg]
