# Supplementary material for: Public and physicians’ support for euthanasia in people suffering from psychiatric disorders: a cross-sectional survey study
Source: BMC Med Ethics. 2019 Sep 11;20:62. doi: 10.1186/s12910-019-0404-8 (PMC6737595; doi:10.1186/s12910-019-0404-8)
Supplement: Supplementary file 2 — Additional file 2: Table S1. Opinions of psychiatrists with regard to EAS in people with psychiatric disorders. (DOCX 18 kb) [file 12910_2019_404_MOESM2_ESM.docx]

Additional file 2: Table S1 Opinions of psychiatrists with regard to EAS in people with psychiatric disorders

|  | (Completely) agree | Do not agree/ disagree | (Completely)  disagree |
| --- | --- | --- | --- |
|  | No. (%) | No. (%) | No. (%) |
| It is impossible to assess whether a psychiatric patient’s suffering is unbearable and without prospect of improvement. | 24 (11.7) | 38 (18.5) | 143 (69.8) |
| A psychiatric patient could suffer unbearably as a result of his or her psychiatric disorder. | 195 (95.1) | 3 (1.5) | 7 (3.4) |
| Providing assistance with suicide is incompatible with a psychiatric care provider relationship. | 35 (17.2) | 38 (18.6) | 131 (64.2) |
| If a patient is able to kill himself or herself in a non-violent manner, he or she should not ask someone else to help. | 28 (13.7) | 36 (17.6) | 141 (68.8) |
| It is impossible to establish whether a wish to die of a psychiatric patient is well-considered or the consequence of an underlying pathology. | 24 (11.7) | 47 (22.9) | 134 (65.4) |
| A psychiatrist, when deciding whether or not to grant a request, need not take account of the solely theoretical possibility that an effective therapeutic treatment might become available in future. | 115 (56.1) | 45 (22.0) | 45 (22.0) |
| It is acceptable for a doctor to provide assistance with suicide in order to prevent suicide. | 82 (40.2) | 68 (33.3) | 54 (26.5) |
| In the case of chronic people with psychiatric disorders, less demanding requirements need to be set for the decisional competence requirement. | 8 (2.9) | 28 (13.7) | 169 (82.4) |

* Number of missings varied between 2 (1.0%) and 3 (1.4%).
